# Supplementary material for: N-Octyl Caffeamide, a Caffeic Acid Amide Derivative, Prevents Progression of Diabetes and Hepatic Steatosis in High-Fat Diet Induced Obese Mice
Source: Int J Mol Sci. 2022 Aug 11;23(16):8948. doi: 10.3390/ijms23168948 (PMC9409300; doi:10.3390/ijms23168948)
Supplement: Supplementary file 1 [file ijms-23-08948-s001.zip › ijms-1860223-supplementary.pdf]

Table S1. Purchase information for antibodies

	Antibody	Catalog No.	Supplier
1	GAPDH	ARG66330	Arigo Biolaboratories Corp., Hsinchu, Taiwan
2	Acetyl-CoA Carboxylase (ACC)	#3676	Cell Signaling Technology, Danvers, MA, USA
3	Phospho-Akt (Ser473)	#9271	
4	Akt	#9272	
5	Phospho-AMPKα (Thr172)	#2535	
6	AMPKα	#2532	
7	β-actin	#3891	
8	Fatty Acid Synthase (FASN)	#3189	
9	Phospho-Glycogen Synthase (Ser641)	#3891	
10	Glycogen Synthase	#3886	
11	Phospho-GSK-3α/β (Ser21/9)	#9331	
12	GSK-3β	#9315	
13	Phospho-IGF-I Receptor β (Tyr1135/1136)/Insulin Receptor β (Tyr1150/1151)	#3024	
14	Insulin Receptor β (IRβ)	#3025	
15	Glucose transporter type 4 (GLUT4)	#2213	
16	Protein-tyrosine phosphatase 1B (PTP1B)	#5311	
17	Perilipin 1	GTX130139	GeneTex, Inc., Irvine, CA, USA
18	Phospho-Acetyl CoA Carboxylase (p-ACC)	#07-303	Merck & Co., Inc., Kenilworth, NJ, USA
19	Peroxisome Proliferator-Activated Receptor gamma (PPAR γ)	MAB3872	
20	Fat specific protein 27 (Fsp27)	NB100-430	Novus Biologicals LCC., Centennial, CO, USA
21	Oxidative tissues-enriched PAT (OXPAT, Perpilin5)	NB110-60509	
22	Sterol Regulatory Element Binding Protein 1c (SREBP1c)	SC13551	Santa Cruz Biotechnology, Inc., Dallas, TX, USA
23	Adipose Differentiation-Related Protein (ADFP Polyclonal, Perpilin2)	PA1-16972	Thermo Fisher Scientific Inc., Rockford, IL, USA
